# Advances in UDP-N-Acetylglucosamine Enolpyruvyl Transferase (MurA) Covalent Inhibition

**DOI:** 10.3389/fmolb.2022.889825

**Published:** 2022-07-20

**Authors:** Maycon Vinicius Damasceno de Oliveira, Renan Machado Furtado, Kauê S. da Costa, Serhii Vakal, Anderson H. Lima

**Affiliations:** ^1^ Laboratório de Planejamento e Desenvolvimento de Fármacos, Instituto de Ciências Exatas e Naturais, Universidade Federal do Pará, Belém, Brazil; ^2^ Institute of Biodiversity, Federal University of Western Pará, Santarém, Brazil; ^3^ Structural Bioinformatics Laboratory, Biochemistry, Faculty of Science and Engineering, Åbo Akademi University, Turku, Finland

**Keywords:** covalent inhibitors, bacterial resistance, fosfomycin, peptidoglycan, MurA enzyme

## Abstract

Peptidoglycan is a cross-linked polymer responsible for maintaining the bacterial cell wall integrity and morphology in Gram-negative and Gram-positive bacteria. The peptidoglycan pathway consists of the enzymatic reactions held in three steps: cytoplasmic, membrane-associated, and periplasmic. The Mur enzymes (MurA-MurF) are involved in a cytoplasmic stage. The UDP-N-acetylglucosamine enolpyruvyl transferase (MurA) enzyme is responsible for transferring the enolpyruvate group from phosphoenolpyruvate (PEP) to UDP-N-acetylglucosamine (UNAG) to form UDP-N-acetylglucosamine enolpyruvate (EP-UNAG). Fosfomycin is a natural product analogous to PEP that acts on the MurA target enzyme via binding covalently to the key cysteine residue in the active site. Similar to fosfomycin, other MurA covalent inhibitors have been described with a warhead in their structure that forms a covalent bond with the molecular target. In MurA, the nucleophilic thiolate of Cys115 is pointed as the main group involved in the warhead binding. Thus, in this minireview, we briefly describe the main recent advances in the design of MurA covalent inhibitors.

## Introduction: MurA Molecular Function

The bacterial peptidoglycan is an extensive mesh-like macromolecule, a cross-linked polymer or a net-like layer, essential for maintaining the bacterial cell wall integrity and morphology (Hsu et al., 2019; Pazos and Peters, 2019; Egan et al., 2020; Pham et al., 2021). Peptidoglycan is present in both Gram-negative and Gram-positive bacteria, being mainly single-layered in diderm bacteria (e.g., *Escherichia coli*) and multilayered in monoderm bacteria (e.g., *Bacillus subtilis*), respectively (Gupta, 2011; Egan et al., 2020; Megrian et al., 2020).

UDP-N-acetylglucosamine enolpyruvyl transferase (MurA) is a cytoplasmatic enzyme from the peptidoglycan pathway responsible for catalyzing the transfer of enolpyruvate from phosphoenolpyruvate to UDP-N-acetylglucosamine to form UDP-N-acetylglucosamine enolpyruvate and release inorganic phosphate (Evangelina et al., 2021). Since the discovery of fosfomycin antibiotic, multiple studies have been carried out on covalent inhibitors (Mihalovits et al., 2019; Hamilton et al., 2020). Recently, the design of new covalent inhibitors has been receiving considerable attention (Mihalovits et al., 2021).

**FIGURE 1:**
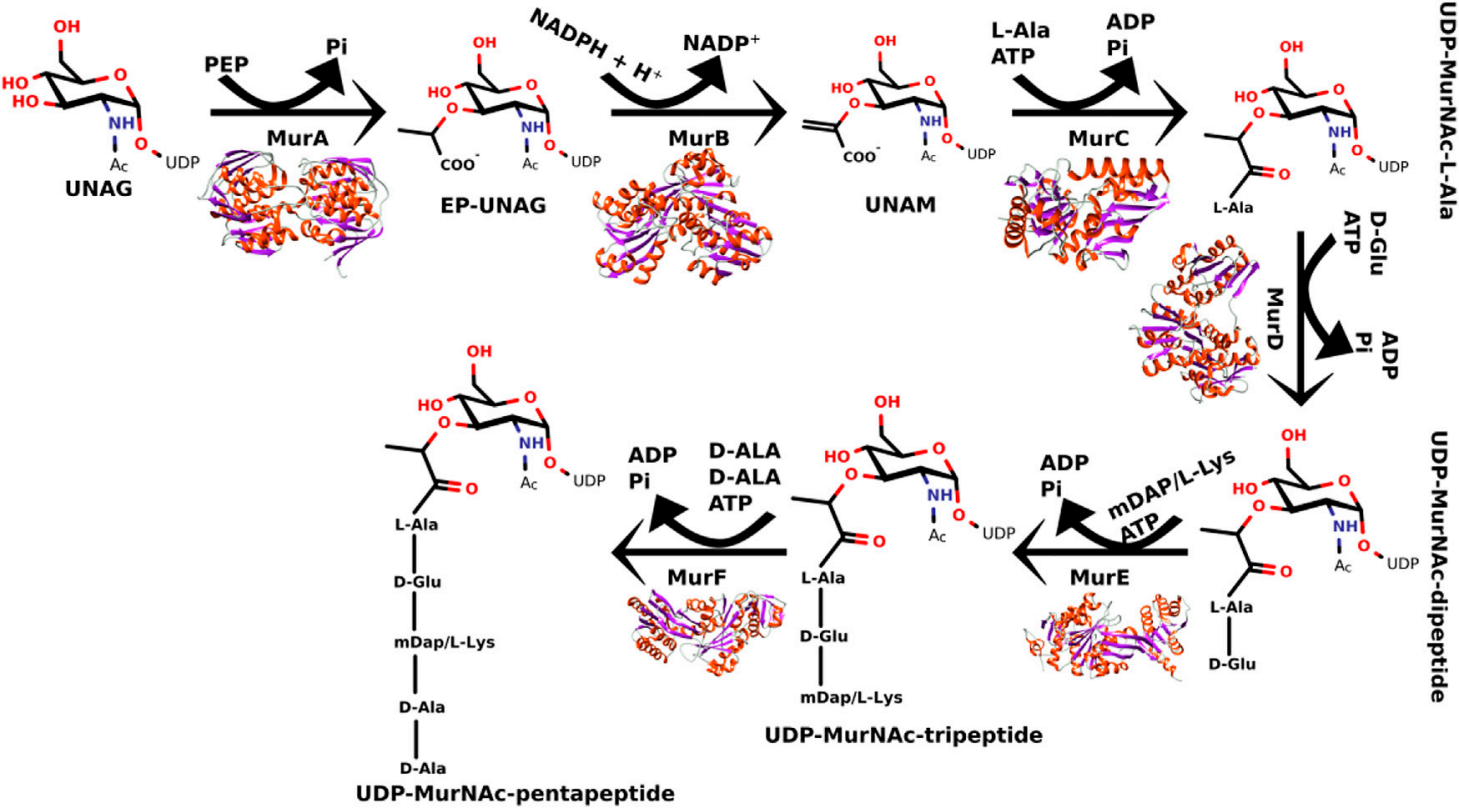
Steps of catalytic reactions performed by the MurA-MurF enzymes in the peptidoglycan biosynthesis.

The peptidoglycan pathway can be divided into three different stages: cytoplasmic, membrane-associated, and periplasmic (Egan et al., 2020). In cytoplasmic stage, the Mur enzyme family (MurA-MurF) is responsible for the conversion of UDP-N-acetylMurAmyl-pentapeptide from UDP-N-acetyl-glucosamine as shown in Figure 1 (Laddomada et al., 2016; Egan et al., 2020).

The MurA enzyme is responsible for transferring enolpyruvate group from phosphoenolpyruvate to UDP-N-acetylglucosamine which leads to the formation of UDP-N-acetylglucosamine enolpyruvate (Kumar et al., 2020). Since the phosphoenolpyruvate substrate acts similarly in other enzymes (e.g., 3-deoxy-D-manno-2-octulosonate-8-phosphate synthase (KDO8PS) (Vainer et al., 2005), 3-deoxy-D-arabino-heptulosonate-7-phosphate synthase (DAHPS) (Burschowsky et al., 2018), 5-enolpyruvylshikimate-3-phosphate synthase (EPSPS) (De Oliveira et al., 2020), and N-acetylneuraminic acid (NeuB) synthase (Popović et al., 2019), it has been shown that analogs of the natural substrate may potentially inhibit MurA (Araújo et al., 2019; De Oliveira et al., 2020). Fosfomycin, cis-1,2-epoxypropyl phosphonic acid, is a MurA inhibitor, which is an antibiotic analogous to PEP used for the treatment of cystitis (Zhanel et al., 2018; Aghamali et al., 2019; Simon et al., 2021).

## MurA and its Covalent Inhibition

Covalent inhibitors can chemically modify the active site of a target protein through covalent binding (Keeley et al., 2018; Martin et al., 2019; Ray and Murkin, 2019). It was believed that covalent inhibitors could pose a high risk to human health due to their toxicity. However, since the last century, a high number of commercially available covalent drugs used to treat various human diseases have been released (Ray and Murkin, 2019; Mihalovits et al., 2021).

**FIGURE 2:**
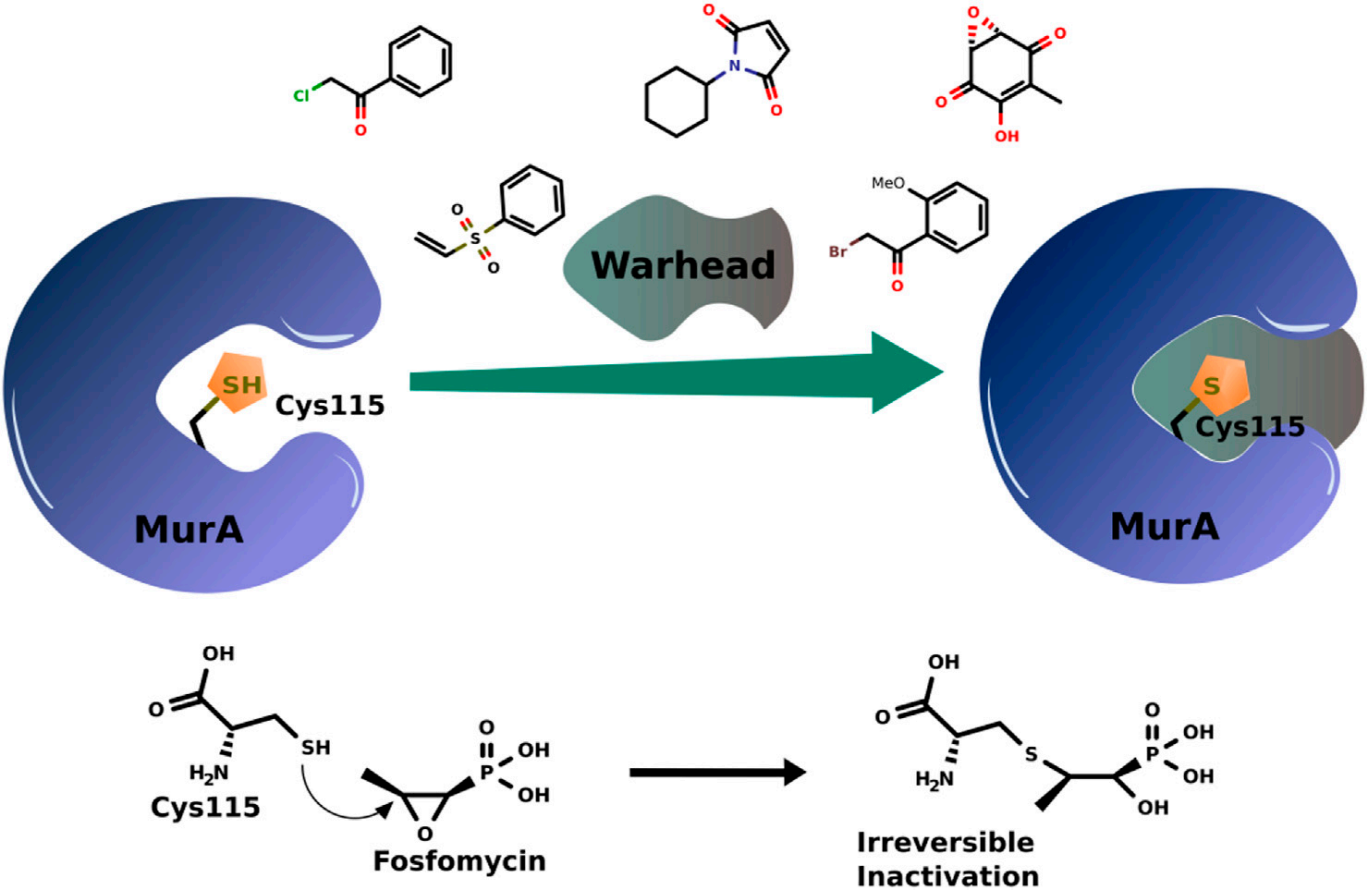
Scheme of covalent bond formation in MurA with some examples of warhead covalent inhibitors and the action mechanism of fosfomycin.

The interaction between a drug and its target through covalent binding can be achieved in two steps: first, a reversible interaction when the inhibitor binds to the protein active site forming the equilibrium bond. In the second step, the warhead covalent inhibitor forms a covalent bond with its target (Ábrányi-Balogh et al., 2018; Sutanto et al., 2020). Among other variables, the strength of this bond governs the reversibility of the complexes formed. In the case of the MurA enzyme, the highly nucleophilic thiolate of Cys115 is utilized to bind the warheads (Figure 2).

Fosfomycin has an oxirane warhead that opens after binding to Cys115 (Figure 2). After years, it is still the most effective and only marketed compound acting on MurA. Thus, the revision presented here aims to give a glimpse of advances in the design of MurA covalent inhibitors.

The problem surrounding fosfomycin, being the only inhibitor commercially available, since its discovery makes increasing number of studies to be carried out to find other covalent inhibitors capable of inhibiting MurA in different types of organisms (Bachelier et al., 2006; Shigetomi et al., 2010; Elsebai et al., 2016; Hamilton et al., 2020; Riyana et al., 2020; Petri et al., 2021). Thus, the enzyme, being one of the main targets of peptidoglycan biosynthesis, has been mostly studied for the development of covalent inhibitors (Table 1).

**TABLE 1:** Some examples of covalent inhibitors against MurA in different organisms, such as *Escherichia coli (E. coli), Enterobacter cloacae (E. cloacae)* and *Staphylococcus aureus (S. aureus)*.

Covalent inhibitors against MurA					
Number ID	2D structure	IUPAC/usual name	IC_50_ (µM)	Organism	Reference
01	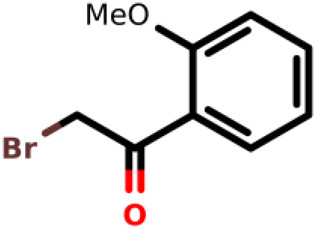	2-bromo-1-(2-methoxyphenyl)Ethan-1-one	0.38	*E. coli*	Mihalovits et al., (2019)
02	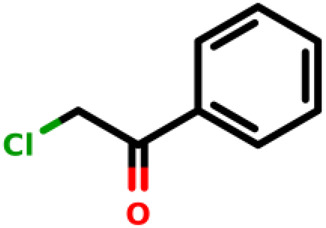	2-chloro-1-phenylethan-1-one	2.25	*E. coli*	Mihalovits et al., (2019)
03	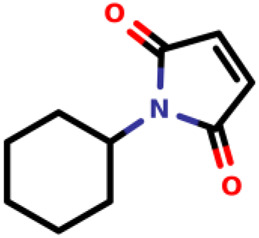	1-cyclohexyl-2,5-dihydro-1H-pyrrole-2,5-dione	0.55	*E. coli*	Mihalovits et al., (2019)
04	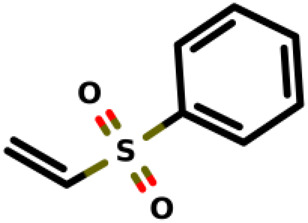	(ethenesulfonyl)benzene	15	*E. coli*	Mihalovits et al., (2019)
05	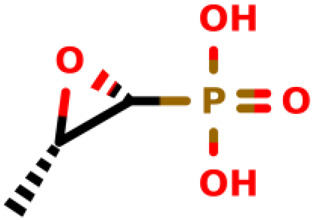	Fosfomycin	8.8	*E. coli*	Baum et al., (2001)
06	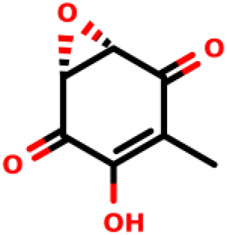	Terreic acid	14	*E. cloacae*	Han et al., (2010)
07	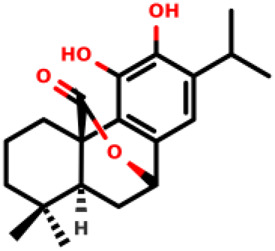	(1R,10S)-3,4-dihydroxy-11,11-dimethyl-5-(propan-2-yl)-16- oxatetracyclo [6.6.2.0^1,10^.0^2,7^]hexadeca- 2 (7),3,5-trien-15-one	2.8 ± 0.7 1.1 ± 0.8	*E. coli* and *S. aureus*	Funes Chabán et al., (2021)
08	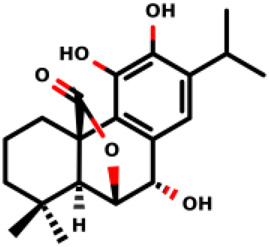	(1R,8S,9S,10S)-3,4,8-trihydroxy-11,11-dimethyl-5-(propan-2-yl)-16- oxatetracyclo [7.5.2.0^1,10^.0^2,7^]hexadeca-2 (7),3,5-trien-15-one	12.9 ± 3.4 5.7 ± 2.1	*E. coli* and *S. aureus*	Funes Chabán et al., (2021)
09	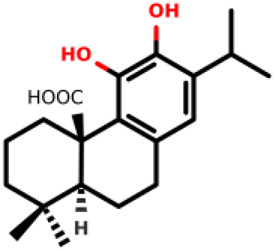	(4aR,10aS)-5,6-dihydroxy-1,1-dimethyl-7-(propan-2-yl)-1,2,3,4,4a,9,10,10a-octahydrophenanthrene-4a-carboxylic acid	25.1 ± 6.5 12.3 ± 2.5	*E. coli* and *S. aureus*	Funes Chabán et al., (2021)
10	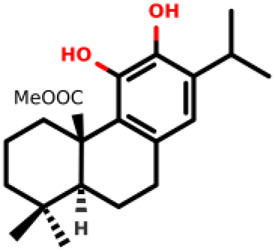	methyl (4aR,10aS)-5,6-dihydroxy-1,1-dimethyl-7-(propan-2-yl)-1,2,3,4,4a,9,10,10a-octahydrophenanthrene-4a-carboxylate	2.8 ± 0.4 3.4 ± 0.3	*E. coli* and *S. aureus*	Funes Chabán et al., (2021)
11	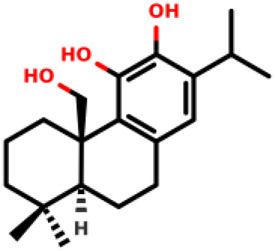	(4bR,8aS)-4b-(hydroxymethyl)-8,8-dimethyl-2-(propan-2-yl)-4b,5,6,7,8,8a,9,10-octahydrophenanthrene-3,4-diol	6.1 ± 0.7 7.4 ± 0.9	*E. coli* and *S. aureus*	Funes Chabán et al., (2021)
12	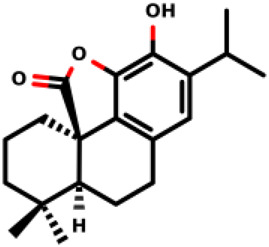	(1R,6S)-12-hydroxy-5,5-dimethyl-11-(propan-2-yl)-14-oxatetracyclo [7.6.1.0^1,6^.0^1,3,16]^hexadeca-9 (16),10,12-trien-15-one	4.8 ± 0.4 7.9 ± 0.6	*E. coli* and *S. aureus*	Funes Chabán et al., (2021)

## Fosfomycin as Food and Drug Administration-Approved Covalent Inhibitor of MurA Enzyme

Fosfomycin, also named monurol or fosfomycin tromethamine, is still the only US Food and Drug Administration-approved (FDA-approved) covalent inhibitor of the MurA enzyme. Since its discovery in 1969, fosfomycin has still been used as a broad-spectrum antibiotic for both Gram-positive and Gram-negative bacteria, and it is currently being used as an alternative agent for the treatment of resistant organisms, such as multidrug-resistant (MDR) bacteria (Falagas et al., 2016; Liu et al., 2020). The structure of fosfomycin contains two key groups: an epoxide and a phosphonic group (Falagas et al., 2019). This antibiotic inhibits the enzymatic reaction catalyzed by MurA involved in the first cytoplasmic step of bacterial wall biosynthesis. The entry of fosfomycin can occur through permeases *via* two pathways, namely, glucose-6-phosphate (G6P) transporter (UhpT) and glycerol-3-phosphate transporter (GlpT) (Castañ;eda-García et al., 2009; Saiprasad and Krishnaprasad, 2016; Díez-Aguilar and Cantón, 2019; Liu et al., 2020). Fosfomycin inhibits MurA by covalent binding to the thiol group of the key cysteine residue (Cys115 in MurA_E.coli_) (Zhanel et al., 2018). Consequently, it blocks the formation of UDP-N-acetylMurAmic acid and interrupts the peptidoglycan biosynthetic pathway.

According to the US FDA, the use of fosfomycin (IUPAC name: [(2R,3S)-3-methyloxiran-2-yl]phosphonate) is allowed only for the treatment of patients with uncomplicated cystitis caused by E. coli and *Enterococcus faecalis* (Baylan, 2010; Silver, 2017). In 1996, this antibiotic was approved for use in acute cystitis treatment in American women (EUA) by single-dose oral therapy of uncomplicated UTIs (Silver, 2017).

Fosfomycin is the unique clinically available inhibitor of MurA acting competitively against phosphoenolpyruvate. Its mode of action is related to the covalent binding to the thiol group of Cys115 residue in the active site of MurA (Kahan et al., 1974; Falagas et al., 2016; Sutanto et al., 2020; Scarpino et al., 2021). Naturally produced by *Streptomyces* spp. (Falagas et al., 2016), fosfomycin is available in drug formulations for oral and intravenous administration. When combined with other drugs, such as amikacin or ceftazidime, it has already been shown to be effective in cases of urinary tract infections (UTIs) with vesicoureteral reflux (VUR) in children (Wu et al., 2016). Treatment with fosfomycin was also effective against patients with UTIs caused by MDR bacterial strains, thus being the first-choice drug for the treatment (Babiker et al., 2019; Tsegka et al., 2022).

Even though terreic acid in *in vitro* tests was shown to be an inhibitor of the MurA enzyme by covalently inactivating it through Cys115 residue, Olsen and co-workers showed *in vivo* that the MurA enzyme is not a molecular target for terreic acid (Olesen et al., 2014). More studies will be necessary for the development of a new antibiotic against the MurA enzyme using the competitive mode of action similar to the fosfomycin.

## Covalent Inhibition of MurA and its Different Classes of Inhibitors

### Hard and Soft Electrophiles

Electrophiles are electron-deficient chemicals that can react with other compounds which have unshared valence electron pairs called nucleophiles (LoPachin et al., 2019). According to Perry’s 1990 Hard and Soft Acids and Bases (HSAB) theory, these electrophilic and nucleophilic species are named according to their polarizability, classified as “soft” or “hard”, or according to the ease of electron density shift to form a covalent bond (LoPachin et al., 2019).

Nowadays, bromo-cyclobutenaminone derivatives are studied as new covalent inhibitors and electrophilic warheads. These inhibitors have an electrophilic character acting as new warheads for the covalent bonding against the Cys115 residue located in the active site of MurA_*E.coli*_ (Hamilton et al., 2020).

The development of a heterocyclic electrophilic fragment library revealed their potential as covalent warheads. This library can be used to identify heterocyclic fragments with significant inhibitory potency against the MurA enzyme (Keeley et al., 2018).

### Michael-Type Nucleophilic Addition

Known as 1,4- or conjugate-addition, or Friedel–Crafts Alkylation is one of the most important nucleophilic additions in the formation of carbon-carbon/carbon-heteroatom bonds in organic synthesis (Saracoglu, 2007). Petri et al. (2021) used the Michael-type nucleophilic addition in 13 covalent fragments that represent various warhead chemotypes. The main compounds based on the IC_50_ value are presented in Table 2. The warhead model 07 (IC_50_ = 13 ± 2.7 µM) forms a covalent bond with Cys115 in the active site of MurA (Petri et al., 2021).

**TABLE 2:** Michael-type nucleophilic addition in main covalent fragments by Petri et al., 2021.

Michael-type nucleophilic addition in main covalent fragments			
R-group	Model ID	Structure	IC_50_ (µM)
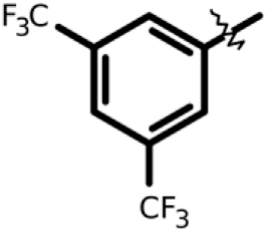	01	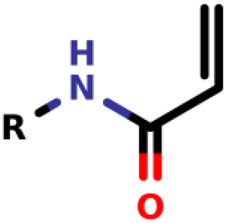	164 ± 14
	02	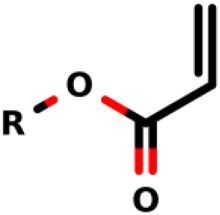	264 ± 23
	03	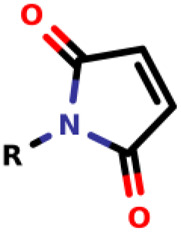	1.5 ± 0.2
	04	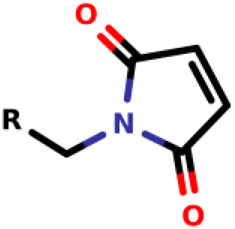	11 ± 2.0
	05	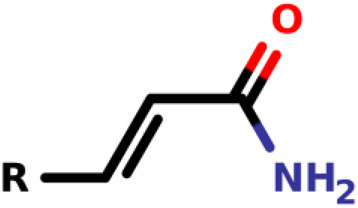	107 ± 11
	06	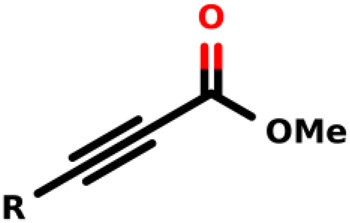	339 ± 31
	07	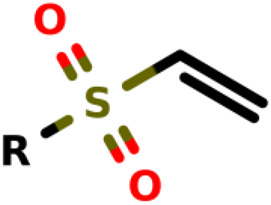	13 ± 2.7
	08	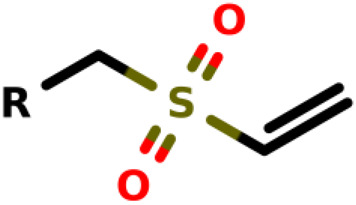	97 ± 10

**FIGURE 3:**
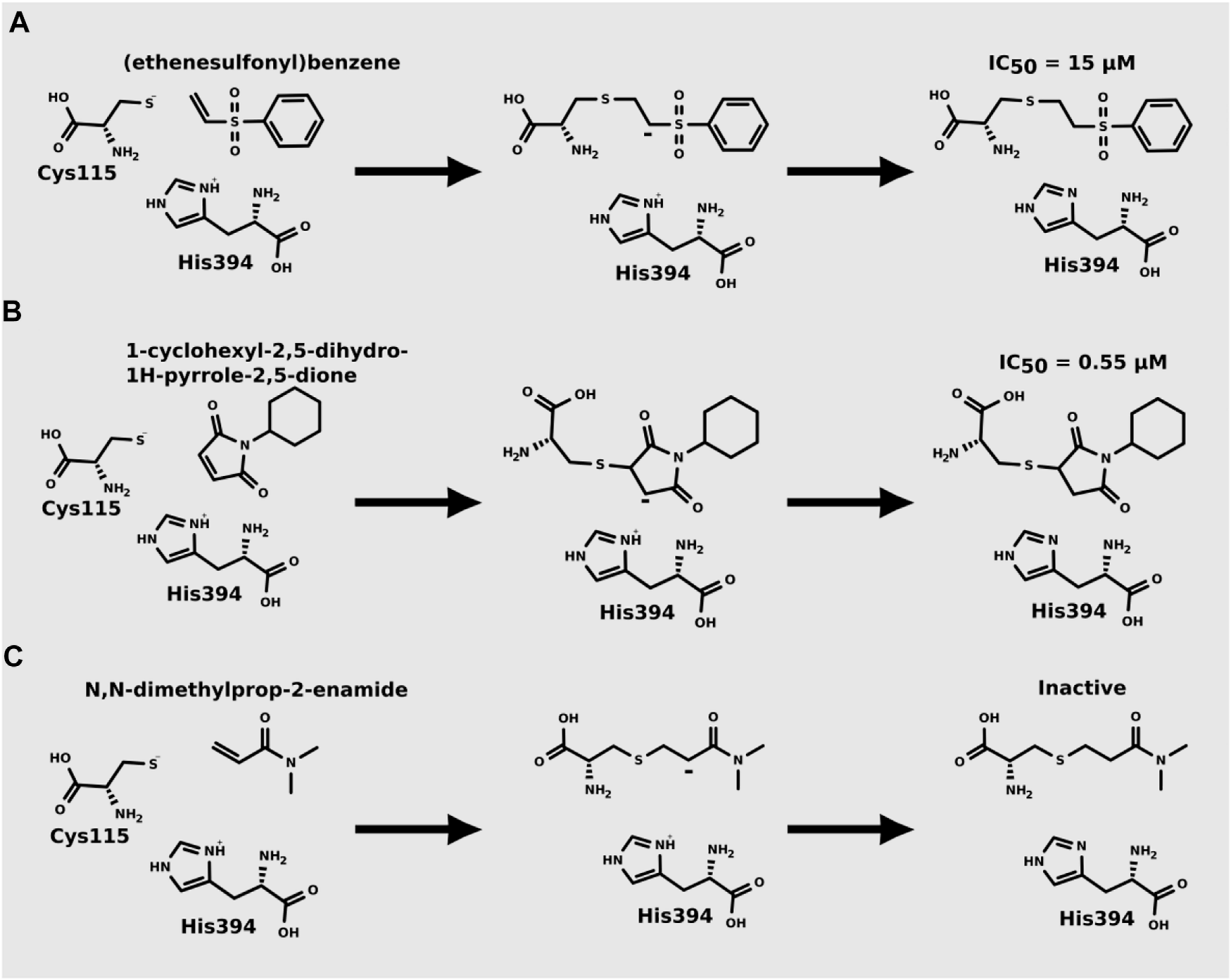
Mechanism for the Michael addition of **(A)** (ethenesulfonyl)benzene **(B)** N,N-dimethylprop-2-enamide, and **(C)** 1-cyclohexyl-2,5-dihydro-1H-pyrrole-2,5-dione against MurA (*E. coli*).

Recently, Mihalovits et al. (2019) showed that some compounds based on the reaction mechanism for the Michael addition and nucleophilic conjugation against MurA from *E. coli* as active inhibitors (01–04 in Table 1) react by binding with Cys115 and interacting with histidine residue (His394) in the MurA binding site (Figure 3). The compound (ethenesulfonyl)benzene is an irreversible inhibitor (IC_50_ = 15 µM), while 1-cyclohexyl-2,5-dihydro-1H-pyrrole-2,5-dione is a reversible inhibitor (IC_50_ = 0.55 μM), and N,N-dimethylprop-2-enamide was shown to be inactive against the *E. coli* MurA enzyme (Mihalovits et al., 2019).

### Natural Products as Covalent Inhibitors of MurA

Natural products obtained from different sources have been widely investigated for the development of new drugs (Do Nascimento et al., 2020). It is believed that 60% of drugs available commercially are derived directly or inspired from natural products (De Cesco et al., 2017; Santana et al., 2021).

Diterpenes, secondary metabolites obtained from plants, and their analogs were explored as inhibitors of the MurA enzyme from *E. coli* and *Staphylococcus aureus* (Funes Chabán et al., 2021)*.* This research showed that six compounds acted as potential inhibitors of MurA in both microorganisms with IC_50_ values between 1.1 and 25.1 µM (07–12 in Table 1). The results revealed that main interactions are made with amino acid residues Arg91, Arg120, and Phe328 (Funes Chabán et al., 2021).

Natural products with antibacterial, antifungal and antiviral activities are often produced by microorganisms as a form of defense (De Cesco et al., 2017). Since 1969, after the discovery of fosfomycin, an increased parcel of natural products that can act as covalent inhibitors against MurA has been studied for the development of new antibiotics. Fosfomycin is a well-known natural covalent inhibitor of the MurA enzyme produced by *Streptomyces sp.* (Kahan et al., 1974). Terreic acid is a covalent inhibitor of MurA *in vitro*, and it is produced by *Aspergillus terreus.* Both compounds inactivate MurA through a similar molecular mechanism of action.

The flavonoid compound catechin from *Uncaria gambir* Roxb. is a potent natural inhibitor of MurA which prevents the growth of *E. faecalis* and *S. sanguinis* (Riyana et al., 2020). Six-tuliposide B is a natural product that shows antibiotic activity against bacterial MurA acting via cnicin mechanism (binding to Cys115 of MurA), and sesquiterpene lactone cnicin also has antibiotic activity (Bachelier et al., 2006; Shigetomi et al., 2010).

Cynaropicrin is a natural product that covalently binds to the thiol group of the Cys115 residue in the MurA active site through Michael addition reaction (Elsebai et al., 2016). This compound is a potent irreversible inhibitor of the MurA enzyme with antibacterial, anti-inflammatory and anti-hepatitis C virus activity (Bachelier et al., 2006; Elsebai et al., 2016).

The structure of several natural products has electrophilic moieties, and they react covalently with nucleophilic functional groups when inserted into their targets (Johansson, 2012). When a compound covalently binds to its molecular target it can be reversible or irreversible. This covalent bond can be generated through several forms of different chemical reactions, for example, disulfide bond, Pinner reaction, or Michael addition (1,4-conjugate addition) (Johansson, 2012).

### Computational Studies of MurA Covalent Inhibitors

Although about twenty articles previously published in the literature describe *in silico* studies of MurA inhibitors, only three of them deal specifically with covalent inhibitors (Dunsmore et al., 2008; Miller et al., 2010), and (Mihalovits et al., 2019).

Dunsmore et al. (2008) identified 2-aminotetralones as a new class of MurA inhibitors that act through the formation of a covalent adduct. Docking of 2-(4-methylpiperazin-1-yl)-1,2,3,4-tetrahydronaphthalen-1-one molecule into *E. coli* MurA showed that keto-group binds close to Cys115 residue and H-bonds with conserved Arg120 residue, which could facilitate thiohemiketal formation and covalent bonding (Dunsmore et al., 2008).

Miller et al. (2010) performed high-throughput screening of a 650 k chemical library against *S. aureus* MurA and identified benzothioxalone derivatives with IC_50_ 0.25–0.51 µM. Docking studies with MurA–UDP-GlcNAc and 5-hydroxy-2H-1,3-benzoxathiol-2-one suggested that thioxalone resides close to the thiol group of the Cys115 residue, which is necessary for covalent adduct formation. Moreover, docking into MurA containing covalently bound fosfomycin fragment showed altered binding mode for benzothioxalone derivatives, in which carbonyl group of thioxalone goes to the secondary pocket, which might explain why some compounds (e.g., compound 18 from this study) can still bind to MurA pre-treated with fosfomycin (Miller et al., 2010).

Mihalovits et al. (2019) performed QM/MM-based molecular dynamics and docking study using six active and three inactive covalent inhibitors of MurA (Mihalovits et al., 2019). These inhibitors can be divided into three groups: oxiranes [fosfomycin, terreic acid, (*S*)-3 (*R*)-3), haloketones (2-bromo-1-(2-methoxyphenyl) etha-1-one, 2-chloro-1-phenylethan-1-one and 2-chloro-N-phenylacetamide), and Michael-acceptors (Figure 3)]. Simulations suggested that the loop closure initiated by UNAG binding brings Cys115 and His394 in a proximity that allows the deprotonation of Cys115 and the formation of the reactive thiolate, which can be involved in various reactions including PEP binding or covalent inhibitor binding. For oxiranes, the key flexible loop can be in a closed (fosfomycin), half-open (fosfomycin) or open (terroic acid) conformation. The reaction was predicted to proceed via a two-step mechanism comprising nucleophilic substitution and subsequent protonation. Haloketones also inhibit MurA via nucleophilic substitution reactions: the formation of the Cys adduct between 2-bromo-1-(2-methoxyphenyl)etha-1-one, 2-chloro-1-phenylethan-1-one and 2-chloro-N-phenylacetamide and the thiolate form of Cys115 proceeds in a single step. For Michael acceptors, the reaction has two steps: first, carbanion intermediate is formed, and then H^+^ transfer between protonated His394 and the negatively charged carbon takes place. Free energy calculations showed that inhibitory activity was more dependent on the energy barrier height of the chemical reaction of covalent binding than on a non-covalent complex formation prior to the chemical reaction.

## Final Considerations

Since its discovery in 1969, fosfomycin remains the only FDA-approved covalent inhibitor used to inhibit the MurA enzyme, being currently available in drug formulations for oral and intravenous administration and being used for the treatment of resistant organisms, such as MDR bacteria. Studies show that fosfomycin combined with other drugs increases the chances of patient cure. Fosfomycin acts through competitive inhibition against PEP, binding the enzyme through the thiol group of Cys115 residue. As the only clinically effective inhibitor available against MurA is a covalent inhibitor, there is an urgent need for more studies to identify molecules that can be used as covalent inhibitors whereas the inhibition of enzyme is a key target for the disruption of the peptidoglycan pathway. Computational methods have been increasingly used in simulations of biological systems, thus, in order to contribute to the development of new drugs, our study demonstrates that new efforts should be made to employ these techniques for the development of MurA covalent inhibitors. Moreover, the present minireview highlightes the recent advances in the development of covalent inhibitors against MurA and the main structural properties associated with its covalent inhibition.
